# Negative-ion field desorption revitalized by using liquid injection field desorption/ionization-mass spectrometry on recent instrumentation

**DOI:** 10.1007/s00216-021-03641-9

**Published:** 2021-09-07

**Authors:** Mathias H. Linden, H. Bernhard Linden, Jürgen H. Gross

**Affiliations:** 1 Linden CMS, Auf dem Berge 25, 28844 Weyhe, Germany; 2 Institute of Organic Chemistry, Heidelberg University, Im Neuenheimer Feld 270, 69120 Heidelberg, Germany

**Keywords:** Field ionization, Field desorption, Liquid injection field desorption/ionization, Field emitter, Negative ions, Ionization process, Anions, Ionic liquids, Cluster ions, Soft ionization, Desorption ionization, Anionic surfactants

## Abstract

Field ionization (FI), field desorption (FD), and liquid injection field desorption/ionization (LIFDI) provide soft positive ionization of gaseous (FI) or condensed phase analytes (FD and LIFDI). In contrast to the well-established positive-ion mode, negative-ion FI or FD have remained rare exceptions. LIFDI provides sample deposition under inert conditions, i.e., the exclusion of atmospheric oxygen and water. Thus, negative-ion LIFDI could potentially be applied to highly sensitive anionic compounds like catalytically active transition metal complexes. This work explores the potential of negative-ion mode using modern mass spectrometers in combination with an LIFDI source and presents first results of the application of negative-ion LIFDI-MS. Experiments were performed on two orthogonal-acceleration time-of-flight (oaTOF) instruments, a JEOL AccuTOF GCx and a Waters Micromass Q-TOF Premier equipped with LIFDI sources from Linden CMS. The examples presented include four ionic liquids (ILs), i.e., *N*-butyl-3-methylpyridinium dicyanamide, 1-butyl-3-methylimidazolium tricyanomethide, 1-butyl-1-methylpyrrolidinium bis(trifluoromethylsulfonyl)imide, and trihexyl(tetradecyl)phosphonium tris(pentafluoroethyl)trifluorophosphate), 3-(trifluoromethyl)-phenol, dichloromethane, iodine, polyethylene glycol diacid, perfluorononanoic acid, anionic surfactants, a tetraphosphazene silanol-silanolate, and two bis(catecholato)silanes. Volatile samples were delivered as vapors via the sample transfer capillary of the LIFDI probe or via a reservoir inlet. Condensed phase samples were applied to the emitter as dilute solutions via the sample transfer capillary. The compounds either yielded ions corresponding to their intact anions, A^−^, or the [M–H]^−^ species formed upon deprotonation. This study describes the instrumental setups and the operational parameters for robust operation along with a discussion of the negative-ion LIFDI spectra of a variety of compounds.

**Supplementary Information:**

The online version contains supplementary material available at 10.1007/s00216-021-03641-9.

## Introduction

Field ionization (FI) and field desorption (FD) are very soft ionization techniques in mass spectrometry that generally deliver intact positive molecular ions, M^+•^, or adduct ions like [M+H]^+^ or [M+alkali]^+^ of molecular analytes [1–4]. In case of ionic compounds, FD and liquid injection field desorption/ionization (LIFDI) spectra reveal the intact cations C^+^, often accompanied by cluster ions [C_n+1_A_n_]^+^.

The first work on negative-ion FI was published by Robertson and Williams in 1964 who reported the production of ions like Cl^−^ from chlorine and tetrachloromethane, I^−^ from iodine, and C_2_H^−^ presumably from hydrocarbon background [5]. The earliest publication on negative-ion FD followed in 1975 by Anbar and St. John [6]. They detected mostly small inorganic anions such as OH^−^, F^−^, Cl^−^, NO_3_^−^, HSO_4_^−^, or BF_4_^−^ from the surface of a freshly broken tungsten rod used as an FD emitter. The inorganic salts were dissolved in polyvinyl alcohol (PVA) serving as the persistent solvent and/or matrix [6].

Negative-ion field ionization (FI) was only realized in 1980 jointly by the groups of Nibbering and Röllgen who achieved M^–•^ ions and 2M^–•^ cluster ions of compounds of high positive electron affinity (*EA*) like tetracyanoethylene and some multiple chlorinated or brominated benzoquinones [7]. They used 10-μm activated tungsten wires as emitters.

In general, however, the inverse FD process that would represent an electron transfer from the emitter to the analyte to yield the electron capture product M^–•^ does not occur, because from standard activated emitters, electrons are emitted below the threshold for negative-ion formation. The resulting electron emission current then causes a spark discharge that leads to the destruction of the emitter. Blank wire emitters and low emitter potentials may avoid such problems [7]. Under these conditions, neutral analytes can form [M–H]^−^ ions or adducts with anions like [M+Cl]^−^ ions [8].

Negative-ion FD of acidic organic compounds delivered [M–H]^−^ and [M+Cl]^−^ ions, respectively, when 10-μm tungsten wires were employed as emitters in combination with polyethylene oxide (PEO 4000) as the highly viscous solvent/matrix [9]. This work was extended to organic sulfonates and carboxylates like dyes and detergents [10, 11]. In this work, the emitter voltages were restricted to about −4 kV and the distance between the emitter and counter electrode was raised up to 8 mm to avoid electron emission, which can be seven orders of magnitude higher than the ion currents [7]. Again, PEO was admixed in excess to serve as a matrix [10]. The salts delivered signals of the anion [A]^−^ and cluster ions of the general composition [C_n–1_A_n_]^−^ [11]. Arginine and sucrose, for example, formed [M–H]^−^ ions under these conditions. The authors also mentioned plans to detect alkylbenzene sulfonates used as surfactants in waste water down to 10^−5^ M concentration [11]. Dähling et al. described the ionization by proton abstraction in negative-ion field desorption mass spectrometry of non-acidic compounds like saccharides and nucleotides from bare or activated tungsten wire emitters in the presence of PEO 4000 [8].

The process of anion formation from solutions in a viscous polymer matrix, polyethylene glycol (PEG 4000) also termed polyethylene oxide (PEO), or from a melt of the neat salt was described as ion desolvation process [12]. Again, essentially fragmentation-free spectra of anionic organic compounds were achieved exhibiting the anion [A]^−^ and [C_n–1_A_n_]^−^ cluster ions [12].

While some more work has been published thereafter [13–15], negative-ion FD-MS has rather remained an exception. Thus, in field desorption mass spectrometry, with less than about ten publications from 1980 to date, negative-ion mode has only played a very minor role, if not to term it exotic. With the advent of fast atom bombardment (FAB) [16, 17], matrix-assisted laser desorption/ionization (MALDI) [18], electrospray ionization (ESI) [19, 20], direct analysis in real time (DART) [21–23], and other ambient MS techniques [21], negative ions have preferably been created by these ionization methods. During the past three decades, the convenience of these newer techniques to deliver negative ions led to a complete neglection of negative-ion FD-MS.

In the light of more recent instrumentation and the improvements of the technique as provided by the advent of LIFDI [24–31], we have revisited negative-ion FD-MS by using the LIFDI setup on two different instrumental platforms. In contrast to the classic implementation of FD, the LIFDI setup offers the additional advantage of sample application to the emitter under the complete exclusion of moisture and air [4, 28, 29, 32–37]. Here, we demonstrate the application of negative-ion LIFDI mode on both a JEOL AccuTOF GCx and a Waters Micromass Q-TOF Premier instrument to a variety of samples.

## Experimental

### Analytes

The compounds used include four ionic liquids (ILs, Merck KGaA, Darmstadt, Germany): *N*-butyl-3-methylpyridinium dicyanamide (**1**), 1-butyl-3-methylimidazolium tricyanomethide (**2**), 1-butyl-1-methylpyrrolidinium bis(trifluoromethylsulfonyl)imide (**3**), and trihexyl(tetradecyl)phosphonium tris(pentafluoroethyl)trifluorophosphate) (**4**). Furthermore, 3-trifluormethyl-phenol (**5**), dichloromethane (**6**), both Sigma-Aldrich (Steinheim, Germany), iodine (**7**, Merck KGaA, Darmstadt, Germany), polyethylene glycol diacid (average molecular weight 600 u) (**8**), perfluorononacoic acid (**9**), both Sigma-Aldrich (Steinheim, Germany), and a dishwashing detergent containing anionic surfactants (Pril Kraftgel, **10**) were used. A sample of solid tetraphosphazene silanol-silanolate (**11**) was obtained from B. Hoge (Bielefeld University, Bielefeld, Germany) and dissolved in dry tetrahydrofuran [38]. Two samples of bis(catecholato)silanes (**12** and **13**) were obtained from L. Greb (Heidelberg University, Heidelberg, Germany) [39]. ILs and solids were delivered to the emitter as solutions at 0.1–0.5 mg ml^−1^, gaseous dichloromethane was admitted via the transfer capillary, and gaseous 3-trifluormethyl-phenol was admitted via the reservoir inlet. All analytes used in this study are compiled in Table 1.

**Table 1:**
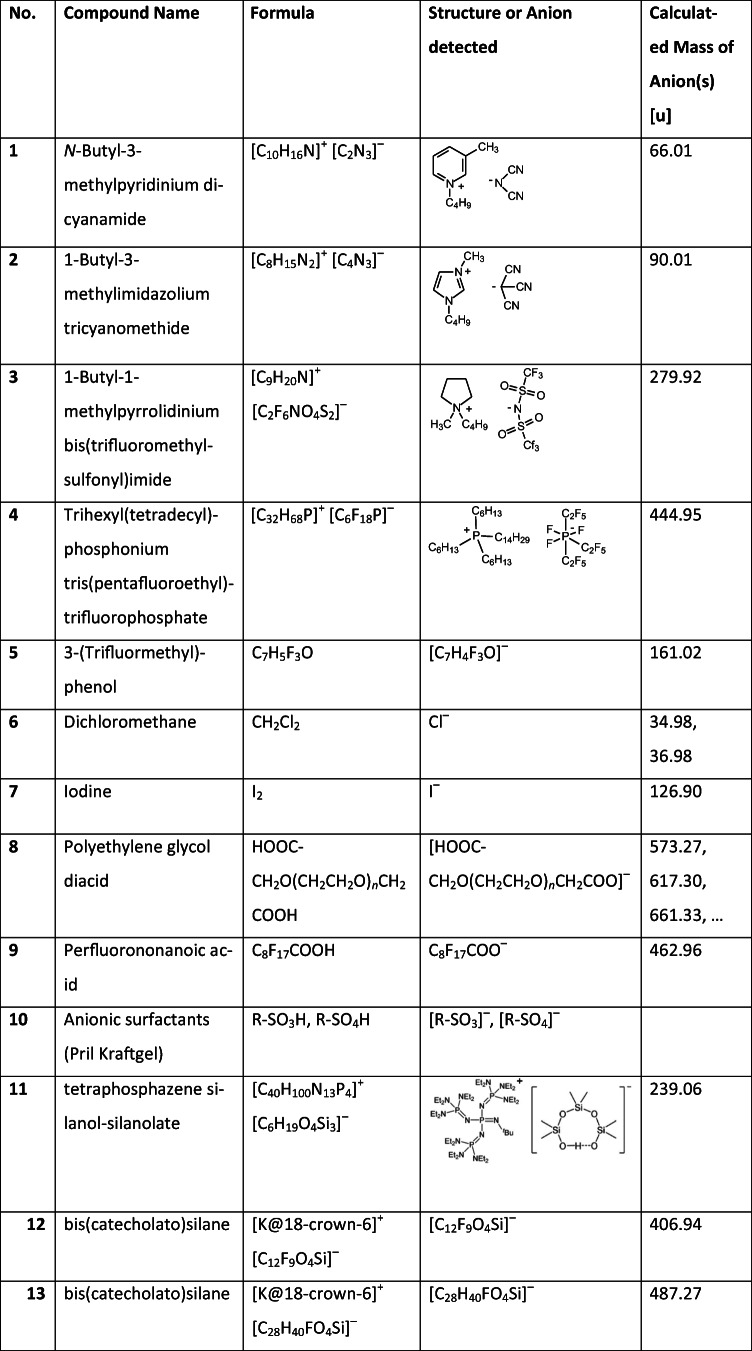
Compilation of compounds studied by negative-ion LIFDI-MS (continued overleaf)

### AccuTOF GCx instrument and LIFDI source

The JEOL AccuTOF GCx orthogonal-acceleration time-of-flight mass spectrometer (Jeol, Tokyo, Japan) was used in combination with an LIFDI source from Linden CMS (Weyhe, Germany); a detailed description of this self-supplied LIFDI source and its operation has recently been published [40]. Emitter high voltage and emitter heating current were controlled using the Linden LIFDI-700 electronics and LIFDI-700 control software. Activated 13-μm tungsten wire emitters either standard type or optimized for negative-ion mode due to strongly suppressed electron emission of whisker tips blunted by thermal deactivation were used. Samples were normally admitted either as vapor or solution via the sample transfer capillary. This fused silica capillary had an inner diameter of 75 μm and a length of 75 cm. The counter electrode was set to a potential of 4.5–6.0 kV. To accelerate the ions generated at the emitter into the ion focusing lens stack of the JEOL instrument, the emitter was set to a potential of −42 V. The settings of the AccuTOF GCx instrument are summarized in Table S1, and for the most part, they just represent the opposite values of those in positive-ion mode [40]. The positioning of the emitter and the ion source potentials were highly reproducible, and thus, only minor adjustments after replacement of an emitter or reinstallation of the source were required. The tuning parameters were saved as a JEOL AccuTOF method file that was loaded and fine-tuned each time after mounting the LIFDI source. Normally, spectra were acquired at a constant emitter heating rate of 30 mA min^−1^ starting at an emitter heating current (EHC) of 0 mA and leading up to 60 mA, in some cases to 80 mA.

External mass calibration was performed in LIFDI mode by measuring a mixture of ILs. The *m*/*z* values of anions and cluster ions were known from previous work [30, 41–44]. Experimental *m*/*z* values are reported here with two digits after the decimal point.

### Waters Micromass Q-TOF Premier and LIFDI source

The original ESI source of a Waters Q-TOF Premier orthogonal-acceleration time-of-flight mass spectrometer (Waters, Manchester, UK) was exchanged for a standard LIFDI source from Linden CMS (Weyhe, Germany). This LIFDI source was essentially identical to that described above for the AccuTOF and was operated analogously except for the potential of the emitter. The same cone potential as used in negative-ion ESI mode was applied to the emitter in negative-ion LIFDI mode in order to keep all other instrumental parameters of the source, flight path, and detector identical to the values of the ESI mode. The instrument control software of the Q-TOF Premier had not to be modified. The settings of the Waters Q-TOF Premier instrument are summarized in Table S2.

Acquisition of negative ions required switching the high voltage of the counter electrode from −10 kV in positive-ion LIFDI mode to +5 kV in negative-ion LIFDI mode. The same geometry was used for either LIFDI polarity without the need to raising the distance between the emitter and counter electrode. Usually, an emitter heating rate of 60 mA min^−1^ was applied up to 120 mA. Typically, ion desorption occurred within 60 s. Within another 60 s, the emitter was heated clean to prevent carry over of samples at the emitter surface. Cation and anion spectra could be acquired from the same vial immediately subsequent to each other (cf. section on “Silanol-silanolate”).

In negative-ion LIFDI mode, mass calibration and instrument fine tuning were performed using the anions A^−^ and the cluster ions [A_2_C]^−^ of a mixture of ionic liquids identical to that described for tuning of the AccuTOF.

## Results and discussion

### Ionic liquids

Ionic liquids (ILs) are involved in various areas of chemistry as they may act as highly polar solvents of extreme thermal stability [45, 46]. Among other techniques, LIFDI has been used for their mass spectral characterization [41]. Moreover, the samples used here have been characterized by direct analysis in real time (DART) where one of the ILs also served as a reference compound for mass calibration [43] as IL cluster ions could be produced beyond *m*/*z* 6500 [44]. Another IL of this set helped in ion source tuning during the development of an LIFDI source for an FT-ICR instrument [30, 31]. Based on this experience, four ILs with different anions were chosen as indicators of anion desorption in negative-ion LIFDI mode (Table 1). The extraordinary thermal stability of ILs and the fact that they form liquid films on the emitter, thereby offering high surface mobility of the anions, [A]^−^, make them ideal candidates to be used in an early phase of testing of an ionization technique. The ILs used here were *N*-butyl-3-methylpyridinium dicyanamide (**1**), 1-butyl-3-methylimidazolium tricyanomethide (**2**), 1-butyl-1-methylpyrrolidinium bis(trifluoromethylsulfonyl)imide (**3**), and trihexyl(tetradecyl)phosphonium tris(pentafluoroethyl)trifluorophosphate (**4**). To effect efficient desorption of the anions, an emitter heating current (EHC) had to be applied. The anions of **1**, **2**, and **4** mainly desorbed in the 35–50-mA EHC range, while that of **3** appeared from 0 to 30-mA EHC.

**Fig. 1:**
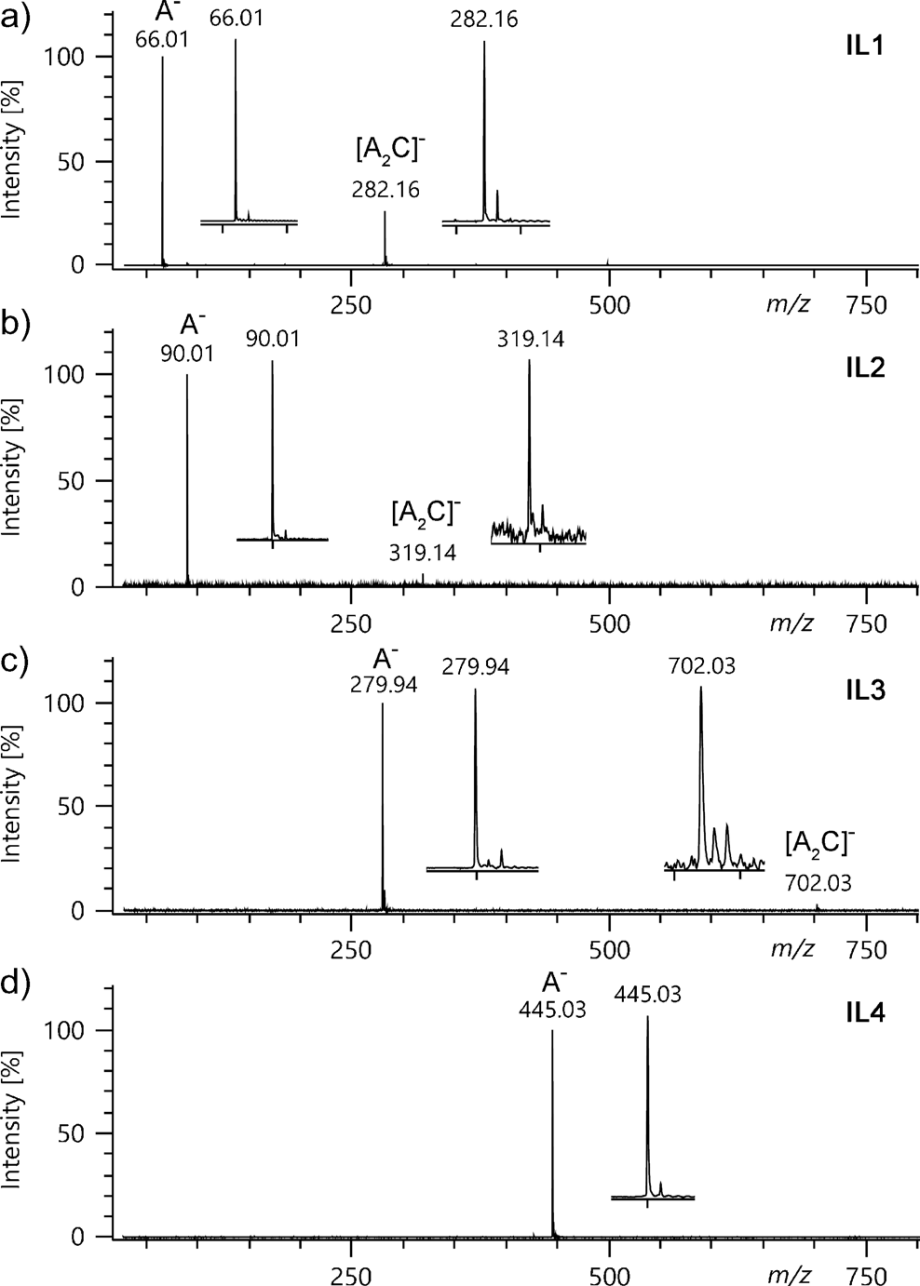
Negative-ion LIFDI spectra of (**a**) *N*-butyl-3-methylpyridinium dicyanamide (**1**), (**b**) 1-butyl-3-methylimidazolium tricyanomethide (**2**), (**c**) 1-butyl-1-methylpyrrolidinium bis(trifluoromethylsulfonyl)imide (**3**), and (**d**) trihexyl(tetradecyl)phosphonium tris(pentafluoroethyl)trifluorophosphate (**4**) as obtained using the JEOL AccuTOF GCx. The ILs yield intensive anion signals. Inserts show the expanded views of the signals of the respective anion, A^−^, and the first cluster ion, [A_2_C]^−^, when present

The negative-ion LIFDI spectra of **1**, **2**, **3**, and **4** show intensive anion signals at *m*/*z* 66.01, *m*/*z* 90.01, *m*/*z* 279.94, and *m*/*z* 445.03 of the respective IL. In addition, the first cluster ions, [A_2_C]^−^, are also observed with the exception of **4** where the cluster ion is out of the *m*/*z* range covered here (Fig. 1). The [A_2_C]^−^ ion of **1** is observed at *m*/*z* 282.16 (cation [C_10_H_16_N]^+^, calc. 150.13 u), of **2** at *m*/*z* 319.14 (cation [C_8_H_15_N_2_]^+^, calc. 139.12 u), and that of **3** at *m*/*z* 702.03 (cation [C_9_H_20_N]^+^, calc. 142.16 u). The inserts of Fig. 1 show the expanded views of the signals of the respective anion, A^−^, and of the first cluster ion, [A_2_C]^−^, when present. In contrast to the other ILs, the anion of **3** contains one sulfur atom that is clearly revealed from the isotopic pattern of A^−^, *m*/*z* 279.94, as is the presence of two sulfur atoms from the isotopic pattern of the corresponding [A_2_C]^−^ ion at *m*/*z* 702.03.

Thus, the negative-ion LIFDI spectra of ILs **1**, **2**, **3**, and **4** demonstrate the basic ability of this instrumental setup to effectively desorb ions from the field emitter and to deliver them to the TOF analyzer.

Analogous results were obtained using the Q-TOF Premier instrument. In the negative-ion LIFDI spectrum of a mixture of 1-butyl-1-methylpyrrolidinium trifluoromethanesulfonate, 1-butyl-1-methylpyrrolidinium bis(trifluoromethylsulfonyl)imide, and trihexyl(tetradecyl)phosphonium tris(pentafluoroethyl)trifluorophosphate, for example, all anions and a cluster ion were observed (Fig. S1).

### Gaseous analytes

In positive-ion FI and FD, toluene or acetone vapor is usually admitted to the ion source to serve for instrument tuning based on a quite constant molecular ion signal in FI operation. While ILs provide very intensive signals, these are more fluctuating in time due to the requirement of emitter heating to generate the anion signal, and moreover, once admitted to the ion source, ILs tend to persist and require several cycles of purging with solvent and emitter baking to disappear. Thus, attempts were made to obtain a constant anion signal by negative-ion FI of a volatile compound that could serve for tuning like toluene or acetone in positive-ion FI mode. Among some halogenated compounds tested, so far, two turned out to deliver a signal that could serve for a basic instrument tuning.

**Fig. 2:**
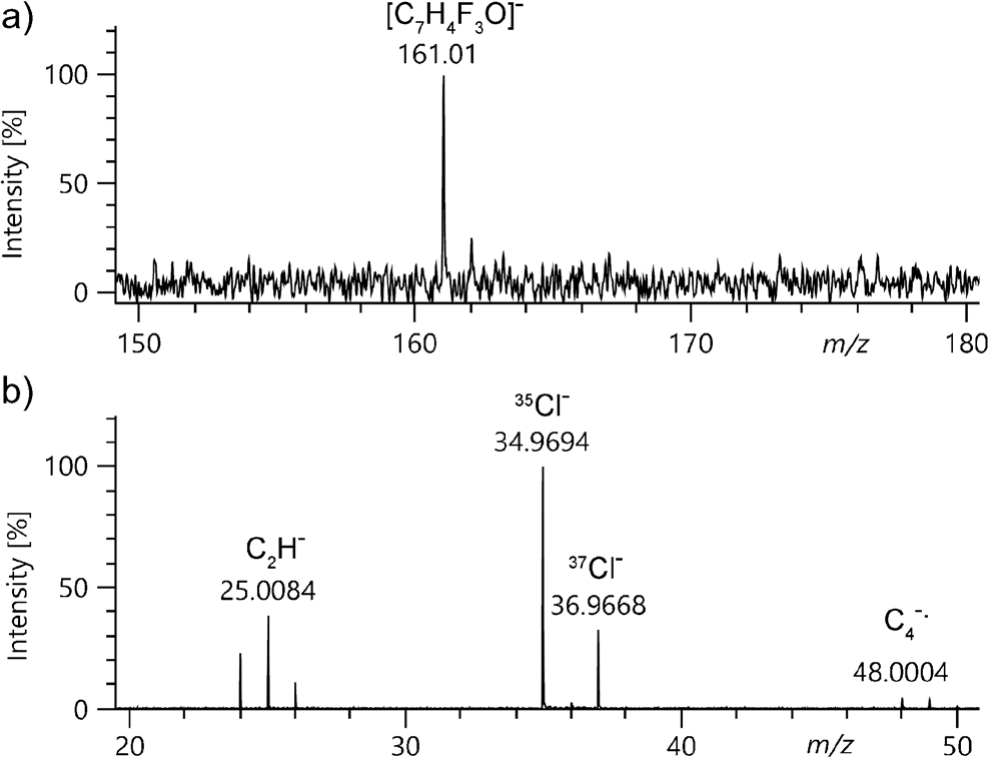
FI mode negative-ion spectra of (**a**) 3-(trifluoromethyl)-phenol (**5**) admitted via the reservoir inlet (80 °C) at an emitter potential of 6.0 kV and (**b**) dichloromethane (**6**) admitted via the sample transfer capillary at an emitter potential of 5.0 kV. Spectra were acquired using the JEOL AccuTOF GCx instrument. In (**a**), the [M–H]^−^ ion at *m*/*z* 161.01 yields a signal of very low intensity with the ^13^C peak just visible and in (**b**), the only signals related to dichloromethane are due to ^35^Cl^−^, *m*/*z* 34.9694 (lock mass), and ^37^Cl^−^, *m*/*z* 36.9668, while the molecular anion or larger fragment ions are absent. Based on the accurate mass, the other signals can be assigned: *m*/*z* 24.0007 to C_2_^–•^, *m*/*z* 25.0084 to C_2_H^−^, *m*/*z* 26.0045 to CN^−^, *m*/*z* 48.0004 to C_4_^–•^, and *m*/*z* 49.0065 to C_4_H^−^

3-(Trifluoromethyl)-phenol (TFP, **5**) was expected to form the deprotonated molecule, [C_7_H_4_F_3_O]^−^. The low volatility of TFP (boiling point 178 °C) did not permit to establish a sufficient sample flow through the sample introduction capillary but required the admission via the heated reservoir inlet at 80 °C. Under these conditions, the [M–H]^−^ ion peak, *m*/*z* 161.01, could be observed at very low intensity (Fig. 2). A signal-to-noise ratio (s/n) of ca. 5 and a barely visible ^13^C isotopic peak rendered this compound not suitable for instrument tuning, however.

Dichloromethane, CH_2_Cl_2_ (**6**), could be admitted via the capillary but also failed to form a molecular anion. Nonetheless, it yielded a ^35^Cl^−^ ion signal, *m*/*z* 34.97, and a ^37^Cl^−^ ion signal, *m*/*z* 36.97 (intensity ratio 3:1, Fig. 2), at a s/n of up to 200. In contrast to positive-ion FI, these signals occurred at almost equal intensity across the 0–80-mA EHC range. The intensity was slightly improved by a stepwise increase of the emitter potential from 5.0 to 5.5 kV. While higher emitter potentials indicated some further improvement, emitter potentials of or above 6.5 kV markedly increased the risk of discharges leading to destruction of the emitter.

Using the peak due to ^35^Cl^−^ as a lock mass for internal mass calibration (calc. *m*/*z* 34.9694), accurate mass could be obtained in this narrow range. Thereby, the additional signals, presumably due to other background in the ion source, could be assigned as follows: *m*/*z* 24.0007 to C_2_^–•^, *m*/*z* 25.0084 to C_2_H^−^, *m*/*z* 26.0045 to CN^−^, *m*/*z* 48.0004 to C_4_^–•^, and *m*/*z* 49.0065 to C_4_H^−^. The occurrence of Cl^−^ and C_2_H^−^ ions, already mentioned in the very first work on negative-ion FI [5], has thus been confirmed.

Even though the chloride ion signals obtained this way are still a bit weak, they may at least serve as a convenient indicator of proper ion source operation and permit some basic tuning prior to admission of samples. Admittedly, there is still some room for improvement in this regard.

### Iodine and a matrix effect

**Fig. 3:**
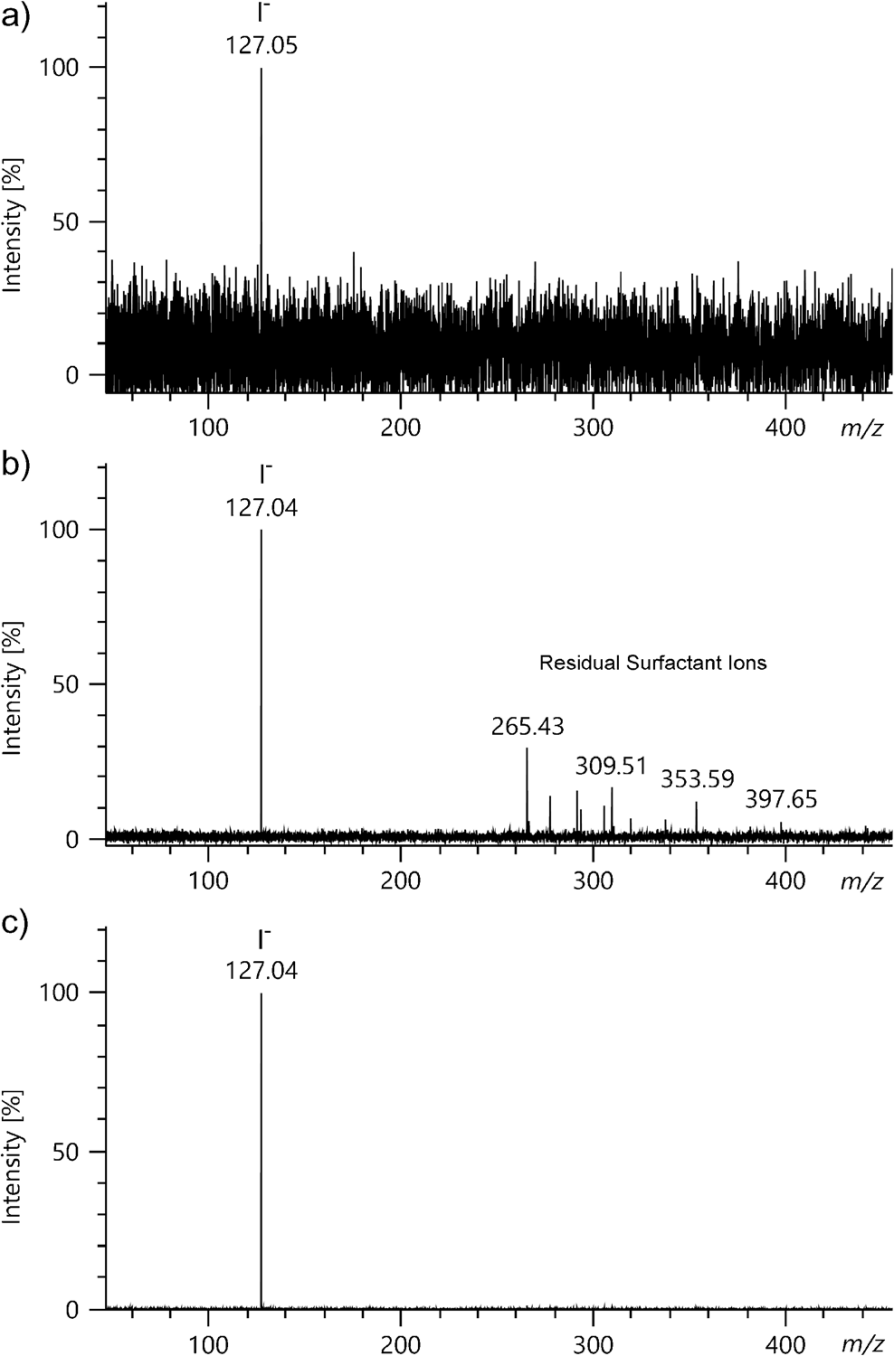
Negative-ion LIFDI spectra of iodine (**7**): (**a**) I_2_ deposited from solution in toluene on a clean emitter, (**b**) I_2_ on an emitter with residual surfactants from a preceding run (peaks at and above *m*/*z* 265.43; cf. Fig. 6), (**c**) I_2_ applied along with PEG 600. The signal-to-noise ratio of the I^−^ signal, *m*/*z* 127.04, improves from (**a**) 3 to (**b**) 40 to (**c**) 400

The low vapor pressure above solid iodine (**7**) did not permit to detect iodide, I^−^, in FI mode when iodine was supplied via the sample transfer capillary as described above for Cl^−^ ion generation from dichloromethane. Nonetheless, I^−^ ions, already mentioned in the very first publication on negative-ion FI [5], could be obtained by applying iodine from solution in toluene to the emitter. However, the signal due to I^−^, *m*/*z* 127.05, showed a poor signal-to-noise ratio of about 3 (Fig. 3). In another run, when a residue of anionic surfactants was present on the emitter (peaks at *m*/*z* 265.43, 309.51, 353.59, and 397.65, cf. section below), the I^−^ ion showed up at s/n = 40, indicating some matrix effect of the residual surfactants. Such matrix effects were already reported in some of the pioneering publications on negative-ion FD [6, 8–12]. Thus, polyethylene glycol 600 (0.1 mg ml^−1^ in methanol) was deposited along with iodine. Under these conditions, the I^−^ peak at *m*/*z* 127.04 improved by another factor of ten exhibiting a s/n of 400 (Fig. 3). This indicates that PEG 600 assisted ion formation in this case. In contrast to the anionic surfactants, PEG 600 did not form any negative ions and therefore did not interfere with the spectrum of iodine. We propose that PEG both serves to avoid rapid sublimation of iodine from the emitter and upon gentle emitter heating forms a thin liquid surface layer that provides analyte mobility on the emitter surface. These findings indicate that in negative-ion FD or LIFDI, the co-deposition of waxy compounds that are unable to form anions themselves can support ion formation and serve to improve spectral quality. This approach can, of course, only be applied when the analyte is compatible with such a compound and therefore is excluded in case of analytes prone to solvolysis.

### Organic acids

Acidic compounds like polyethylene glycol diacid (**8**) and perfluorononanoic acid (PFNOA, **9**) can be expected to readily form anions by deprotonation. Therefore, these compounds were selected for further testing of negative-ion LIFDI mode.

**Fig. 4:**
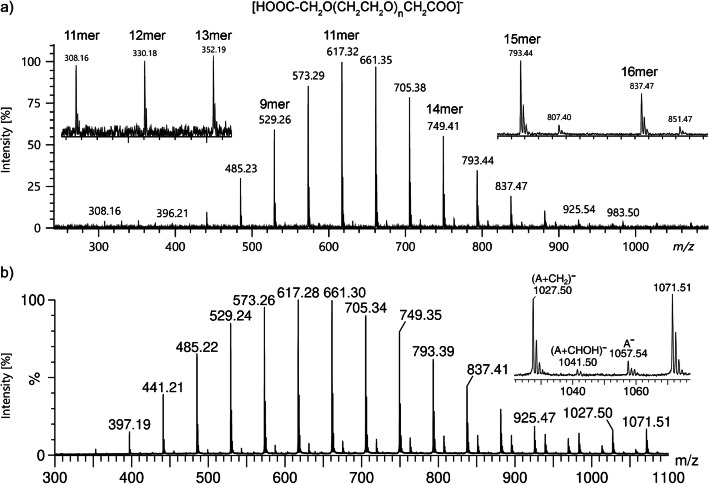
Negative-ion LIFDI spectra of polyethylene glycol diacid (**8**) using (**a**) a JEOL AccuTOF GCx and (**b**) a Waters Q-TOF Premier. In (**a**), the [HOOC-CH_2_O(CH_2_CH_2_O)_*n*_CH_2_COO]^−^ ions range from the 7mer to 18mer. The insert on the left shows an expanded view of some [M–2H]^2−^ ions occurring at low intensity and the right insert shows the isotopic patterns of the [M–H]^−^ ions of the 15mer and 16mer, respectively. In (**b**), the same main series of anions is detected from 5mer to 18mer together with a series of [A+CH_2_]^−^ in the 5 to 12mer range and [A+CHOH]^−^ in the 10 to 35mer range (shown up to the 22mer for better comparison)

Polyethylene glycol diacid, HOOC-CH_2_O(CH_2_CH_2_O)_*n*_CH_2_COOH (average molecular weight 600 u), also yielded [M–H]^−^ ions under negative-ion DART conditions. Thus, this particular sample had been characterized by DART-FT-ICR-MS before [47]. The ion observed in the negative-ion LIFDI spectrum at *m*/*z* 617.32, for example, can thus be correlated to the [M–H]^−^ ion of the 11mer, [C_26_H_49_O_16_]^−^ (calc. 617.3026, Fig. 4a). Obviously, the main series of ions observed here belongs to [HOOC-CH_2_O(CH_2_CH_2_O)_*n*_CH_2_COO]^−^ ions ranging from the 7mer at *m*/*z* 441.21 to the 18mer at *m*/*z* 925.54. In addition, some [M–2H]^2−^ ion signals occur in the *m*/*z* 250–450 range at up to 5% relative intensity; e.g., the 11mer is also reflected by the [M–2H]^2−^ ion peak at *m*/*z* 308.16. The spectrum shown was acquired by summation of the ions produced in the 35–50-mA EHC range.

In addition to the same main series, two additional series of ions appear in the spectrum acquired with the Waters Q-TOF Premier (Fig. 4b). The first additional series, presumably [A+CH_2_]^−^, ranges from the 5mer to the 12mer and the second series, tentatively assigned to [A+CHOH]^−^ ions, from the 10mer to the 35mer.

**Fig. 5:**
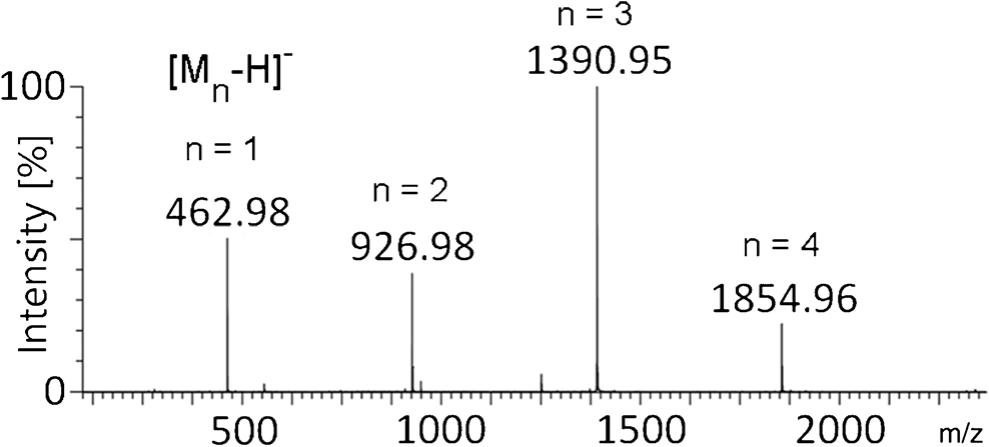
Negative-ion LIFDI spectrum of perfluorononanoic acid (**9**) as acquired by the Waters Q-TOF Premier mass spectrometer. The spectrum exhibits the intact [M–H]^−^ ion, [C_8_H_17_COO]^−^, *m*/*z* 462.98, and three [M_n_–H]^−^ cluster ions (*n* = 2–4) at *m*/*z* 926.98, 1390.95, and 1854.96, respectively

The surfactant perfluorononanoic acid (PFNOA, **9**) is used for the production of polyvinylidene fluoride. Its negative-ion LIFDI spectrum exhibits the [M–H]^−^ ion, i.e., [C_9_F_17_O_2_]^−^, *m*/*z* 462.98 (calc. *m*/*z* 462.96) by deprotonation of the molecule plus [M_n_–H]^−^ cluster ions ranging from *n* = 2 through *n* = 4 at *m*/*z* 926.98 (calc. *m*/*z* 926.93), *m*/*z* 1390.95 (calc. *m*/*z* 1390.90), and *m*/*z* 1854.96 (calc. *m*/*z* 1854.87), respectively (Fig. 5).

### Surfactants from dishwashing detergent

**Fig. 6:**
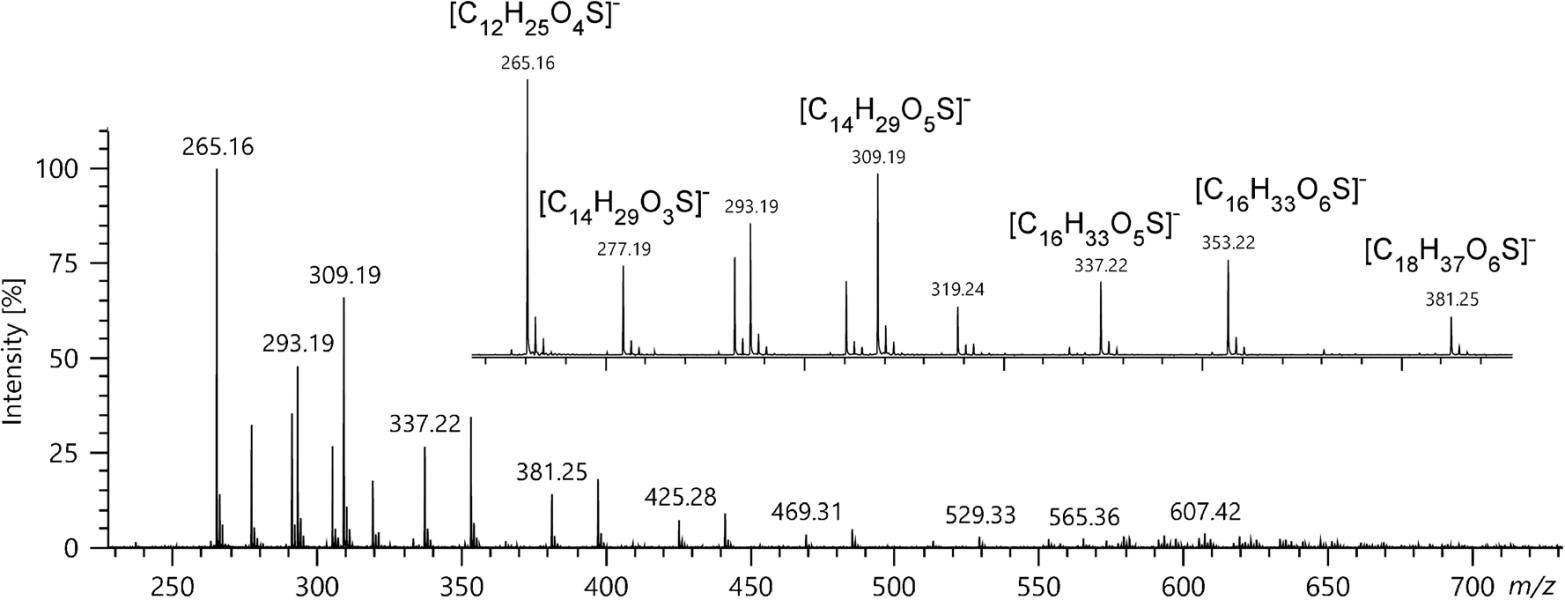
Negative-ion LIFDI spectrum of a dishwashing detergent as obtained using the JEOL AccuTOF GCx instrument. Intensive signals corresponding to various alkylsulfates and alkylsulfonates are observed. The spectrum represents the sum of ions desorbed in the 47–59-mA heating current range, and while acquired covering a range *m*/*z* 30–1200, it just shows the *m*/*z* 230–730 range as no peaks were observed outside this range. The insert shows an expanded view of the *m*/*z* 260–390 range to reveal the isotopic patterns

Household dishwashing detergents are known to contain anionic surfactants. Thus, the negative-ion LIFDI spectrum of a dishwashing detergent (Pril Kraftgel, **10**) was acquired to explore the range of applications [11]. The negative-ion LIFDI spectrum as obtained by using the JEOL AccuTOF GCx instrument is shown in Fig. 6. Desorption of anions did mainly occur across the heating current range of 47–59 mA. The displayed range is limited to *m*/*z* 230–730 as no peaks above 0.5% rel. int. were observed outside this range even though a range of *m*/*z* 30–1200 was acquired. The intensive signals were tentatively assigned to homologous series of various alkylsulfates and alkylsulfonates. To provide an independent proof of this assumption, the negative-ion electrospray spectrum was acquired using the Bruker ApexQe FT-ICR mass spectrometer (Fig. S2). Overall, the negative-ion ESI spectrum exhibited better s/n ratio and revealed a wider distribution of surfactant ions than the negative-ion LIFDI spectrum, probably due to the fact that in LIFDI notable emitter heating was required to desorb the ions. Based on the ESI-FT-ICR data, the ionic formulas could be assigned to series of alkylsulfates and alkylsulfonates with different additional functional groups (Figs. S2 and S3). For example, the signal at *m*/*z* 265.16 reflects the saturated alkylsulfate [C_12_H_25_O_4_S]^−^, the signal at *m*/*z* 277.19 corresponds to the saturated alkylsulfonate [C_14_H_29_O_3_S]^−^, and the peak at *m*/*z* 309.19 assigned to [C_14_H_29_O_5_S]^−^ represents a variant having one oxygen atom more than an alkylsulfate, e.g., by hydroxylation, or two oxygen atoms more than an alkylsulfonate, e.g., by hydroxylation and/or ether groups in the chain. The spacing between peaks belonging to a homologous series, e.g., *m*/*z* 265.16, 309.19, and 353.22, indicates C_2_H_4_O monomer units. However, no attempt was made to identify all compound classes in this mixture beyond this basic identification serving as a control of the LIFDI data.

### Silanol-silanolate

**Fig. 7:**
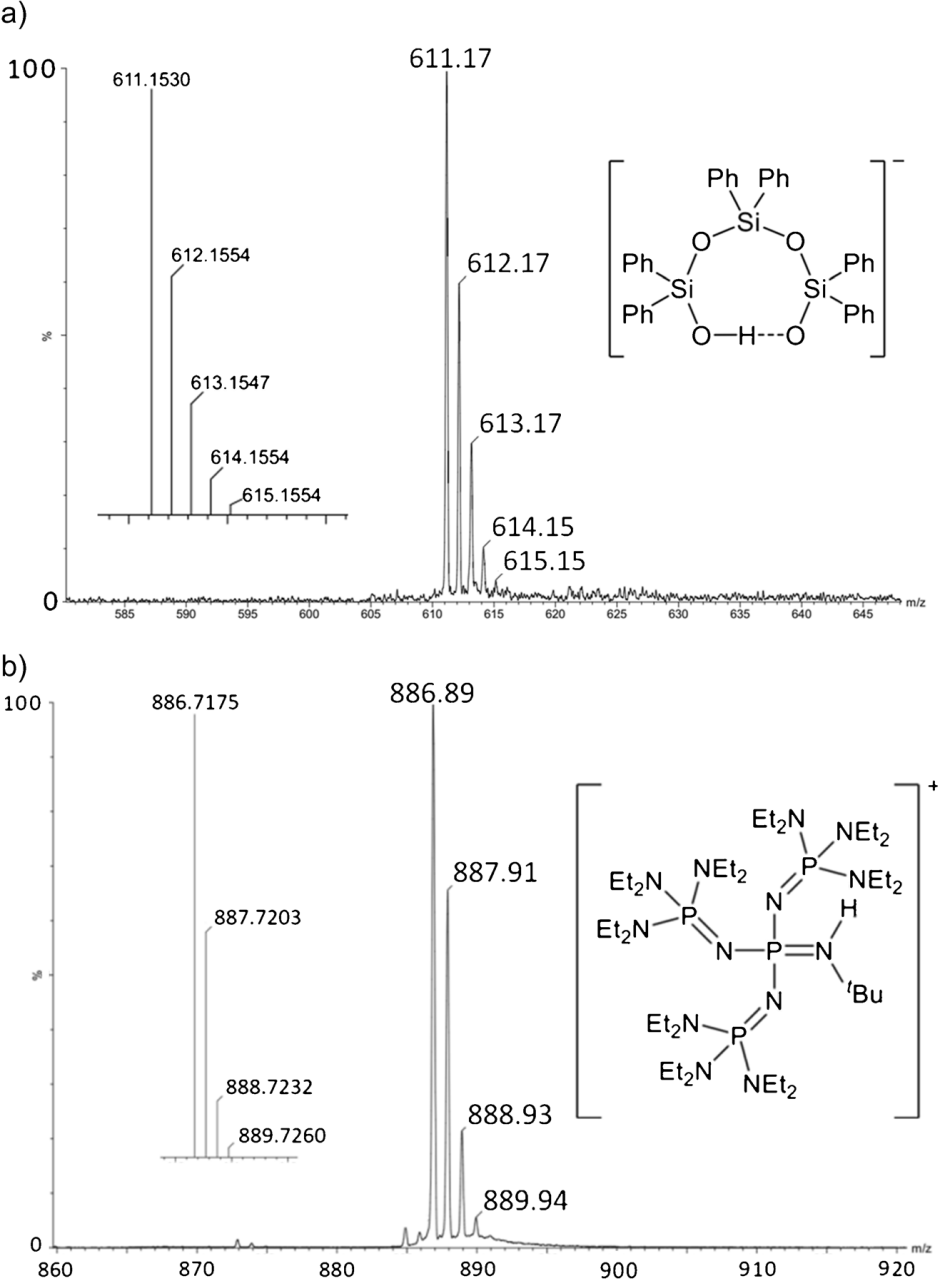
LIFDI spectra of (**a**) the novel silanol-silanolate anion [C_36_H_31_O_4_Si_3_]^−^ and (**b**) its weakly coordinating phosphazenium counter ion [C_40_H_100_N_13_P_4_]^+^. The structures of both ions and their calculated isotopic patterns are also shown

Handling of silanol-sinalolates requires the strict exclusion of moisture to avoid decomposition of these compounds, and thus, mass spectral characterization of the silanolate ion as well as elemental analysis of the ion pair was still missing, because the ion pair could neither be characterized by elemental analysis nor by any ionization technique in mass spectrometry so far [38]. Thus, a solution of tetraphosphazene silanol-silanolate (**11**) [C_40_H_100_N_13_P_4_]^+^ [C3_6_H_31_O_4_Si_3_]^−^, anion *m*/*z* 611.15 (calc.), was prepared in dry THF and was analyzed by negative-ion as well as positive-ion LIFDI-MS using the Waters Micromass Q-TOF instrument (Fig. 7). The spectra show the intact anion and intact cation, respectively. Both spectra were acquired immediately after each other by reversing the ion source polarity. To exclude any air during the measurement, the entrance of the LIFDI capillary remained in the headspace of the capped septum vial during the measurements.

### Bis(catecholato)silanes

**Fig. 8:**
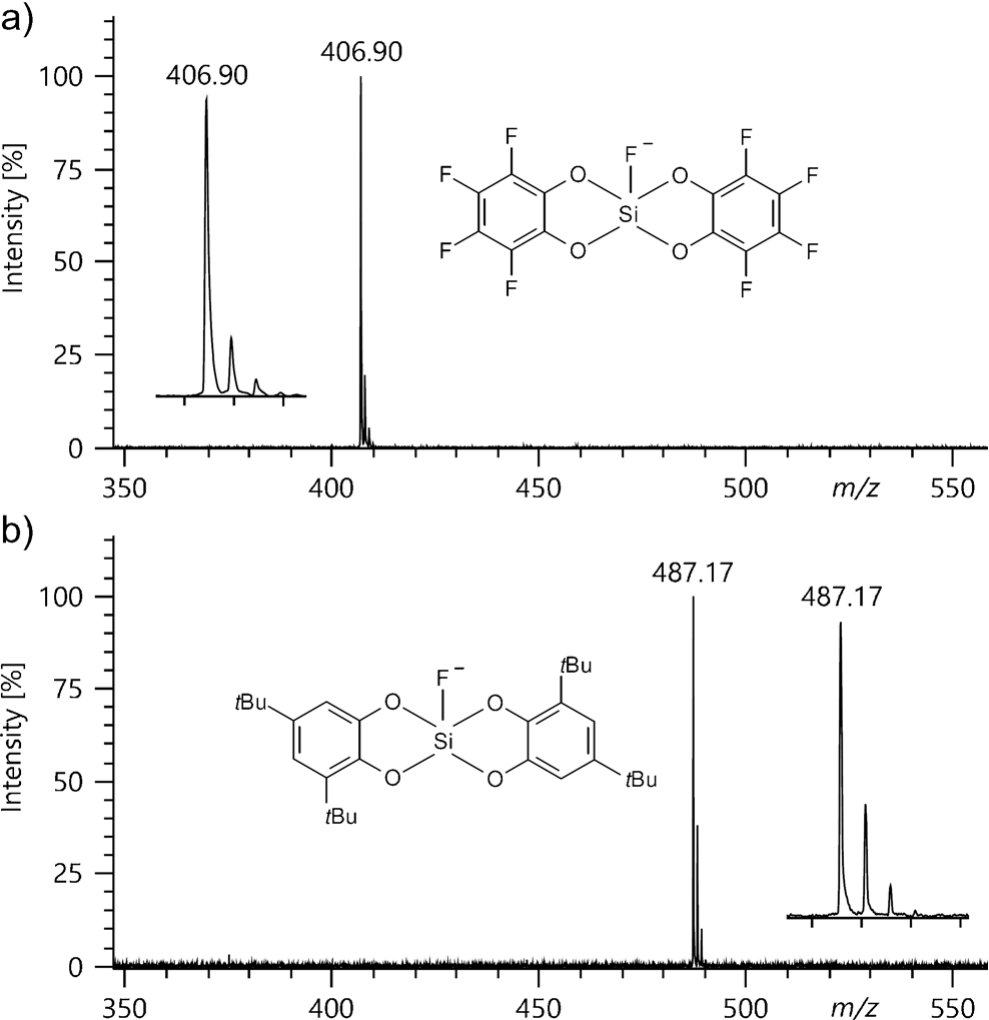
Negative-ion LIFDI spectra of (**a**) [K@18-crown-6]^+^ [C_12_F_9_O_4_Si]^−^ (**12**) and (**b**) [K@18-crown-6]^+^ [C_28_H_40_FO_4_Si]^−^ (**13**). Spectrum (**a**) represents the sum of ions formed in the 25–40-mA EHC range and (**b**) is from the 40–50-mA EHC range. Both were obtained with standard LIFDI emitters and at a counter electrode potential set to 5.0 kV. Insert show expanded views of the isotopic patterns of the respective anions

The two bis(catecholato)silanes (**12** and **13**) represent examples of silicon Lewis superacids introduced only recently [39]. These compounds are sensitive to moisture and are thus typical candidates for LIFDI-MS. To measure the negative-ion LIFDI spectra of [K@18-crown-6]^+^ [C_12_F_9_O_4_Si]^−^ (**12**) and [K@18-crown-6]^+^ [C_28_H_40_FO_4_Si]^−^ (**13**), the samples were supplied as solutions in dry dichloromethane and analyzed using the JEOL AccuTOF GCx instrument. At a counter electrode potential of 5.0 kV and with standard LIFDI emitters, both compounds yielded spectra with clear signals corresponding to the respective anions, i.e., *m*/*z* 406.90 due to [C_12_F_9_O_4_Si]^−^ (calc. *m*/*z* 406.94) and *m*/*z* 487.17 corresponding to [C_28_H_40_FO_4_Si]^−^ (calc. *m*/*z* 487.27, Fig. 8).

## Conclusion

Modern TOF mass spectrometers provide tremendously improved sensitivity and speed of analysis as compared with the magnetic sector instruments employed in the 1980s. Combined with the advancement of LIFDI over the classic FI and FD modes, negative-ion analysis can now be achieved much more efficiently. As demonstrated here, negative-ion LIFDI mode delivers promising results with a variety of samples that are either ionic or prone to negative-ion formation. In LIFDI mode, the compounds either delivered ions corresponding to their intact anions (ILs, silanol-silanolate, bis(catecholato)silanes) or the [M–H]^−^ species formed upon deprotonation. Due to dissociative ionization, dichloromethane only yielded chloride ions.

The spectra have been obtained on two different instrumental platforms, thereby indicating that the application is not restricted to a single highly specific setup. Rather, any mass spectrometer that can be operated in negative-ion mode and be equipped with an LIFDI source will be able to deliver this type of analysis. One may expect that negative-ion LIFDI mode will soon provide a highly useful addition to the mass spectral toolbox, in particular in the field of the analysis of anionic compounds sensitive to air and moisture. We are continuing to investigate this field of applications.

## Supplementary information

ESM 1 (PDF 1689 kb)
