# Supplementary material for: Negative-ion field desorption revitalized by using liquid injection field desorption/ionization-mass spectrometry on recent instrumentation
Source: Anal Bioanal Chem. 2021 Sep 7;413(27):6845–55. doi: 10.1007/s00216-021-03641-9 (PMC8551092; doi:10.1007/s00216-021-03641-9)

Negative-Ion Field Desorption Revitalized by Using Liquid Injection Field Desorption/Ionization-Mass Spectrometry on Recent Instrumentation

Mathias H. Linden[1], H. Bernhard Linden[1], Jürgen H. Gross[2]*

[1] Linden CMS, Auf dem Berge 25, 28844 Weyhe, Germany

[2] Institute of Organic Chemistry, Heidelberg University, Im Neuenheimer Feld 270
69120 Heidelberg, Germany,

* Send correspondence to Jürgen H. Gross
email juergen.gross@oci.uni-heidelberg.de
phone +49/6221/54-8409

Supporting Information

Table S1: Typical instrument tuning parameters for the JEOL AccuTOF GCx in negative-ion LIFDI operation.

**Basic Settings**		**Advanced Settings**	
Ion Chamber	70 °C	Ion Source	–35 V
Reservoir	80 °C	Reflectron	–1150 V
GC Interface	90 °C	Push	–777.8 V
Repeller	–1.5 V	Pull	777.8 V
Lens 1	1000 V	Suppress	–0.20 V
Lens 2	680 V	Flight Tube	7000 V
Lens 3	80 V		
Slit Lens	50 V	**Acquisition**	
Deflector	50 V	Sampling Interval	0.5 ns
Lens 2 Balance	10 V	Recording Interval	1.00 s
Deflector Balance	6.0 V	Accumulation Time	0.950 s
Push Bias	0.90 V	Wait Time	0.050 s
Detector	2820 V	*m/z*	30-1200
**Vacuum**			
Ion Source	1.2–2.0 x 10^{-3} Pa		
Analyzer	2.0–4.0 x 10^{-5} Pa		

Table S2: Typical instrument tuning parameters for the Waters Q-TOF Premier in negative-ion LIFDI operation.

**Voltages and Temperatures**			
Ion Chamber	ambient temperature	Ion Source	–10 V
Sampling Cone	–10 V	Reflectron	–2160 V
Extraction Cone	0 V	Pusher	–905 V
Ion Guide	1 V	Puller	630 V
Pre-Filter	10 V	Pusher Offset	–1 V
Acceleration 1	80 V	Flight Tube	9100 V
Acceleration 2	200 V		
Aperture	70 V	**Acquisition**	
Transport 1	60 V	Sampling Interval	0.64 ns
Transport 2	60 V	Recording Interval	1.00 s
Steering	0 V	Accumulation Time	0.980 s
Tube Lens	75 V	Wait Time	0.020 s
Detector	650 V	*m/z*	50–1800
**Vacuum**			
Ion Source	2.0–5.0 x 10^{-4} Pa		
Analyzer	1.5–3.0 x 10^{-5} Pa		

Fig. S1: Negative-ion LIFDI spectrum of a mixture of ionic liquids as obtained using the Q-TOF Premier instrument. The compounds used here are 1-butyl-1-methylpyrrolidinium trifluoromethanesulfonate, 1-butyl-1-methylpyrrolidinium bis(trifluoromethylsulfonyl)imide, and trihexyl(tetradecyl)phosphonium tris(pentafluoroethyl)trifluorophosphate. The first cluster ion of 1-butyl-1-methylpyrrolidinium bis(trifluoromethylsulfonyl)imide is also observed. The Inserts show the expanded views of the signals of the respective anions (profile spectra on the right) together with their calculated isotope pattern (bar graphs on the left).

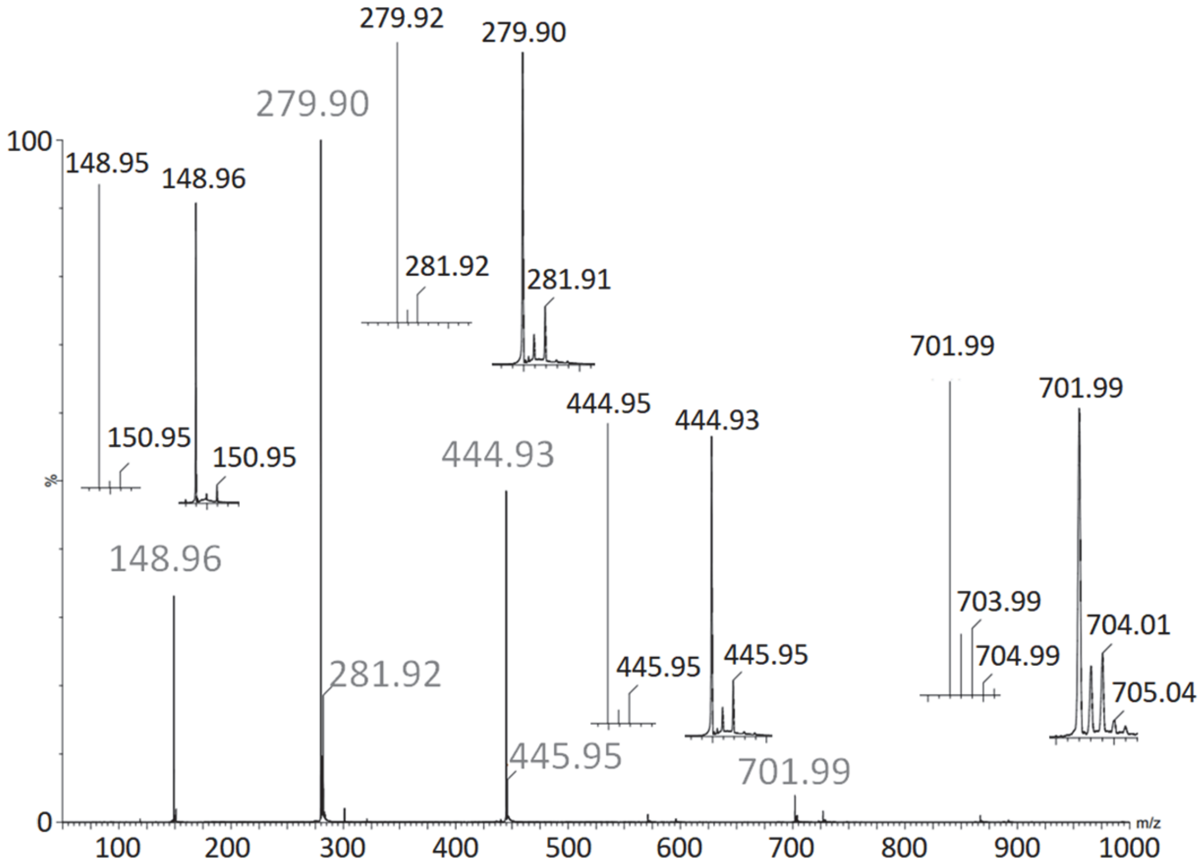

Fig. S2: Negative-ion electrospray spectrum of the dish washer Pril Kraftgel in MeOH : H2O = 9 : 1 as obtained on the Bruker ApexQe FT-ICR mass spectrometer. Mass accuracy is better than 2 ppm. Peaks with formula assignment are marked with an orange dot. The ionic formulas can be assigned to series of alkylsulfates and alkylsulfonates with different additional functional groups.

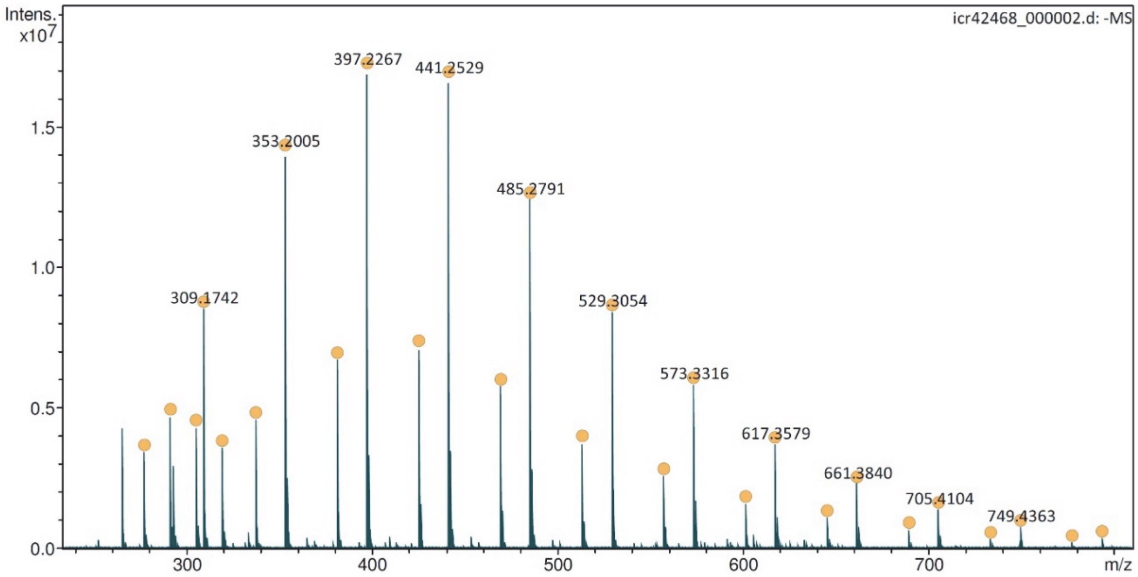

Meas. m/z	Ion Formula	m/z	err [ppm]	mSigma	rdb	e$^-$ Conf	N-Rule
265.1480	C12H25O4S	265.1479	-0.3	9.7	0.5	even	ok
277.1844	C14H29O3S	277.1843	-0.4	8.8	0.5	even	ok
291.2000	C15H31O3S	291.1999	-0.3	4.8	0.5	even	ok
305.2157	C16H33O3S	305.2156	-0.4	8.3	0.5	even	ok
309.1742	C14H29O5S	309.1741	-0.3	8.0	0.5	even	ok
319.2314	C17H35O3S	319.2312	-0.4	10.0	0.5	even	ok
337.2056	C16H33O5S	337.2054	-0.5	11.2	0.5	even	ok
353.2005	C16H33O6S	353.2003	-0.4	10.0	0.5	even	ok
381.2318	C18H37O6S	381.2316	-0.4	11.4	0.5	even	ok
397.2267	C18H37O7S	397.2265	-0.4	11.3	0.5	even	ok
425.2580	C20H41O7S	425.2578	-0.4	10.2	0.5	even	ok
	C26H35NO4	425.2572	-2.0	41.7	10.0	odd	ok
441.2529	C20H41O8S	441.2528	-0.3	13.1	0.5	even	ok
	C26H35NO5	441.2521	-1.9	49.3	10.0	odd	ok
	C27H39NS2	441.2529	0.1	53.7	9.0	odd	ok
469.2843	C22H45O8S	469.2841	-0.6	22.8	0.5	even	ok
	C28H39NO5	469.2834	-2.0	55.8	10.0	odd	ok
	C29H43NS2	469.2842	-0.2	56.5	9.0	odd	ok
485.2791	C28H39NO6	485.2783	-1.7	52.5	10.0	odd	ok
	C29H43NOS2	485.2792	0.1	56.5	9.0	odd	ok
513.3106	C31H47NOS2	513.3105	-0.3	56.6	9.0	odd	ok
	C30H43NO6	513.3096	-1.9	57.5	10.0	odd	ok
529.3054	C30H43NO7	529.3045	-1.7	43.0	10.0	odd	ok
	C31H47NO2S2	529.3054	-0.1	71.9	9.0	odd	ok
557.3369	C32H47NO7	557.3358	-1.9	50.6	10.0	odd	ok
	C33H51NO2S2	557.3367	-0.3	68.6	9.0	odd	ok
573.3316	C32H47NO8	573.3307	-1.6	35.4	10.0	odd	ok
	C33H51NO3S2	573.3316	-0.1	65.7	9.0	odd	ok
601.3629	C34H51NO8	601.3620	-1.6	56.9	10.0	odd	ok
	C35H55NO3S2	601.3629	-0.1	74.9	9.0	odd	ok
617.3579	C35H55NO4S2	617.3578	-0.2	71.5	9.0	odd	ok
645.3888	C37H59NO4S2	645.3891	0.4	100.4	9.0	odd	ok
661.3840	C37H59NO5S2	661.3840	0.1	71.8	9.0	odd	ok
	C51H49	661.3840	-0.0	116.7	27.5	even	ok
689.4153	C39H63NO5S2	689.4153	0.0	108.4	9.0	odd	ok
	C53H53	689.4153	-0.0	152.0	27.5	even	ok
705.4104	C39H63NO6S2	705.4102	-0.2	87.1	9.0	odd	ok
	C53H53O	705.4102	-0.3	136.7	27.5	even	ok
733.4418	C41H67NO6S2	733.4415	-0.3	99.6	9.0	odd	ok
	C55H57O	733.4415	-0.4	119.9	27.5	even	ok
749.4363	C41H67NO7S2	749.4364	0.2	105.8	9.0	odd	ok
	C55H57O2	749.4364	0.1	138.7	27.5	even	ok
777.4677	C43H71NO7S2	777.4677	0.0	127.5	9.0	odd	ok
	C57H61O2	777.4677	-0.0	176.2	27.5	even	ok
793.4631	C43H71NO8S2	793.4627	-0.5	63.4	9.0	odd	ok
	C57H61O3	793.4626	-0.6	118.6	27.5	even	ok

Fig. S3: Expanded view of the signals in the range *m/z* 397.0–399.3 of a negative-ion electrospray spectrum of the dish washer Pril Kraftgel in MeOH : H2O = 9 : 1 as obtained on the Bruker ApexQe FT-ICR mass spectrometer. The formula assignments to alkylsulfates and alkylsulfonates (previous figure) is supported by the doublet peak at *m/z* 399 and the Δ(*m/z*) = 1.9959 value (calc. Δm ^{32}S to ^{34}S = 1.9958 u) between the peak of the monoisotopic ion, *m/z* 397.2267, and the one corresponding to an ion with ^{34}S at *m/z* 399.2226 that proves the presence of sulfur. Analogous isotopic patterns were observed at the other signals.

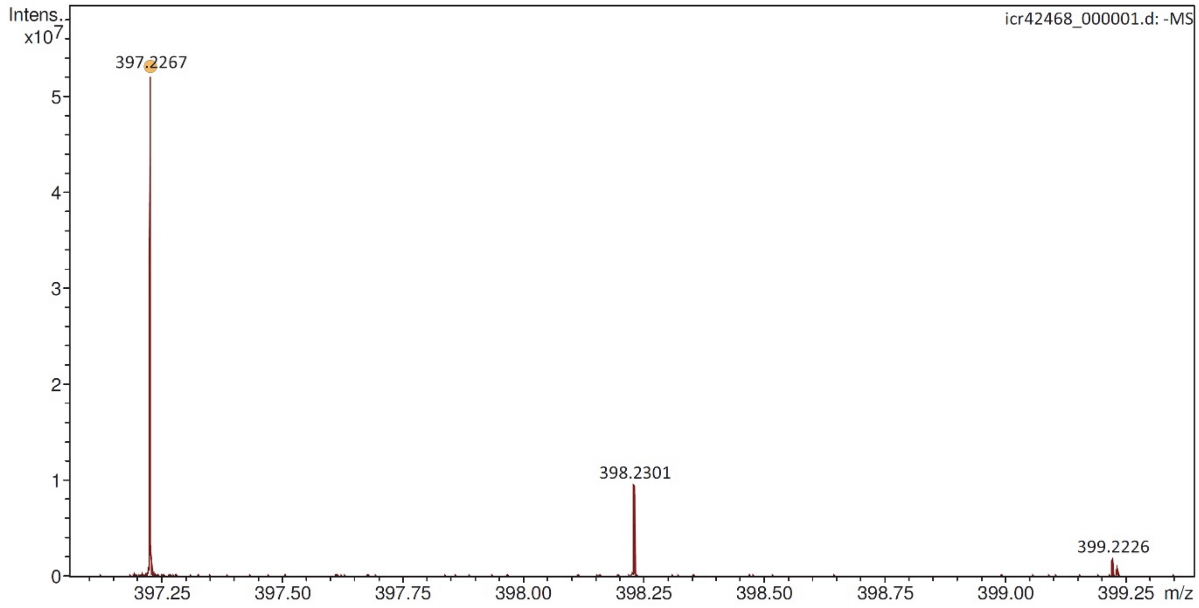

Supplement: Supplementary file 1 — (PDF 1689 kb) [file 216_2021_3641_MOESM1_ESM.pdf]
